# Effects of non-pharmacological interventions on inflammatory biomarker expression in patients with fibromyalgia: a systematic review

**DOI:** 10.1186/s13075-015-0789-9

**Published:** 2015-09-26

**Authors:** Kenji Sanada, Marta Alda Díez, Montserrat Salas Valero, María Cruz Pérez-Yus, Marcelo M P Demarzo, Mauro García-Toro, Javier García-Campayo

**Affiliations:** Aragon Health Sciences Institute (IACS), Zaragoza, Spain; Department of Psychiatry, Showa University School of Medicine, Tokyo, Japan; The Primary Care Prevention and Health Promotion Research Network (REDIAPP), Barcelona, Spain; Department of Psychiatry, Miguel Servet University Hospital, University of Zaragoza, Avda Isabel La Católica I., 50009 Zaragoza, Spain; Department of Preventive Medicine, Federal University of Sao Paulo (UNIFESP), “Mente Aberta” - Brazilian Centre for Mindfulness and Health Promotion, Sao Paulo, Brazil; Research Institute of Health Sciences (IUNICS), University of Balearic Islands, Palma, Spain

## Abstract

**Introduction:**

Fibromyalgia (FM) is a prevalent disorder. However, few studies have evaluated the effect of treatment interventions on biomarker expression. The aim of this review was to explore the efficacy of non-pharmacological interventions on inflammatory biomarker expression, specifically cytokines, neuropeptides and C-reactive protein (CRP), in FM patients.

**Method:**

A literature search using PubMed, EMBASE, PsycINFO and the Cochrane library was performed from January 1990 to March 2015. Randomized controlled trials (RCTs) and non-RCTs published in English, French or Spanish were eligible.

**Results:**

Twelve articles with a total of 536 participants were included. After exercise, multidisciplinary, or dietary interventions in FM patients, interleukin (IL) expression appeared reduced, specifically serum IL-8 and IL-6 (spontaneous, lipopolysaccharide (LPS)-induced, or serum). Furthermore, the changes to insulin-like growth factor 1 (IGF-1) levels might indicate a beneficial role for fatigue in obese FM patients. In contrast, evidence of changes in neuropeptide and CRP levels seemed inconsistent.

**Conclusion:**

Despite minimal evidence, our findings indicate that exercise interventions might act as an anti-inflammatory treatment in FM patients and ameliorate inflammatory status, especially for pro-inflammatory cytokines. Additional RCTs focused on the changes to inflammatory biomarker expression after non-pharmacological interventions in FM patients are needed.

## Introduction

Fibromyalgia (FM) is a prevalent disorder that affects to 2–8 % of the population [1–3], and it is characterized by widespread pain, fatigue, memory problems and sleep disturbances. Currently, it is one of the most common disorders seen by primary care physicians [4] and the second most common rheumatic disorder after osteoarthritis [5].

There are many possible treatments for FM that can be classified as pharmacological and non-pharmacological, and the latter can also be subclassified into psychological and non-psychological interventions [6–12]. The efficacy of these treatments is considered low to moderate, and there are no significant differences between them when administered in primary care or specialized settings [13].

The main criteria assessed in pharmacological and non-pharmacological trials are function, quality of life, pain, depression, anxiety and fatigue. All of these are subjective symptoms assessed by questionnaire and are rated by the patient or the clinician. However, few trials on FM include biological outcomes, in particular an assessment of inflammatory biomarkers, despite neurogenic neuroinflammation having been associated with the pathogenesis of FM [14]. As mentioned, non-pharmacological treatments are systematically recommended as an adjunctive treatment for FM [10]; therefore our aim was to determine the efficacy of non-pharmacological interventions on inflammatory biomarkers in FM patients, specifically cytokines, neuropeptides, and C-reactive protein (CRP).

## Methods

This systematic review followed the Preferred Reporting Items for Systematic Reviews and Meta-Analyses guidelines (PRISMA) [15].

### Search strategy and data extraction

A comprehensive computerized literature search of PubMed, EMBASE, PsycINFO and the Cochrane library was performed from January 1990 to March 2015 by an expert in this field (MSV). The starting date was established because the American College of Rheumatology (ACR) criteria for the classification, with the definition of FM, were published in 1990 [16]. For example, the following search terms for the PubMed database were used: (“fibromyalgia” [MeSH Terms] OR “fibromyalgia” [All Fields] OR “Fatigue Syndrome, Chronic” [Mesh]) AND (“Cytokines” [Mesh] OR “Interleukins” [Mesh] OR “Biological Markers” [Mesh] OR “Neuropeptides” [Mesh] OR “C-Reactive Protein” [Mesh] OR biomarker* OR cytokine* OR interleukin* OR neuropeptide* OR “C-reactive protein” OR CRP).

The reference lists of the identified original articles and reviews were also searched manually for additional studies. The literature search was conducted independently by two authors (KS and MCPY). Any disagreements were resolved by discussion and consensus, and when in doubt, the final decision was made in consultation with a third author (JGC). The last search was conducted on 13 March 2015.

### Eligibility criteria

The study eligibility criteria are shown in Table 1. We excluded studies from the following cases: the first trial was conducted in patients not only with FM but also with chronic fatigue syndrome (CFS) (e.g., Light et al. [17]); the second excluded trial used biomarkers as predictors or related factors to symptom severity (e.g., Ross et al. [18]); the third assessed a mixed type treatment plan with pharmacological and non-pharmacological intervention [19]; the fourth conducted exercise tests [20–22]; the fifth was written in German [23]; and the sixth was published as a letter [24].

**Table 1:** Study eligibility criteria

	Inclusion criteria	Exclusion criteria
Participants	Fibromyalgia patients (FM) or FM and a healthy population	Patients with other diseases, mixed types of patients (i.e., FM patients with other disorders), only a healthy population
	No restrictions on the number of participants and the diagnostic procedures were applied	
Biomarkers	Cytokines, neuropeptides and CRP	Other biomarkers
Interventions	Non-pharmacological interventions were eligible	Mixed or blended non-pharmacological interventions, only pharmacological interventions
Outcome	At least one biomarker (cytokine or neuropeptide or CRP) outcome	Studies in which biomarkers were used as predictors to identify the participants were excluded
Study design	RCTs, Non-RCTs	
Publications	Published in English, French or Spanish and as full-text articles in peer-reviewed scientific journals from January 1990 to March 2015	Published in other languages and as reviews, case reports or letters

*CRP* C-reactive protein, *Non-RCTs* non-randomized controlled trials, *RCTs* randomized controlled trials

### Assessment of study quality

The risk-of-bias tool is generally fitted to randomized controlled trials (RCTs), but we can apply it to non-RCTs, and a specific tool for this use is under development [25]. In this systematic review, the risk of bias in the included studies was assessed using the Cochrane risk-of-bias tool [25].

## Results

**Fig. 1:**
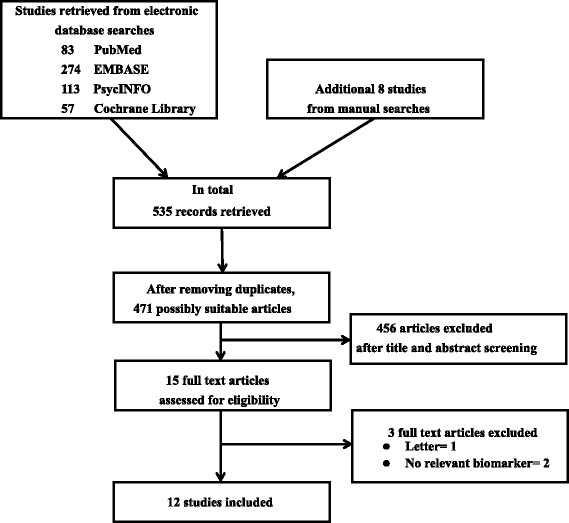
Algorithm for study selection (following Preferred Reporting Items for Systematic Reviews and Meta-Analysis guidelines, Shamseer et al., [15])

After the initial search of 535 records, 64 were found to be duplicates (Fig. 1). We screened the titles and abstracts, and 15 articles were assessed as full text. We finally included 12 articles with a total of 536 participants.

### Characteristics of included studies

**Fig. 2:**
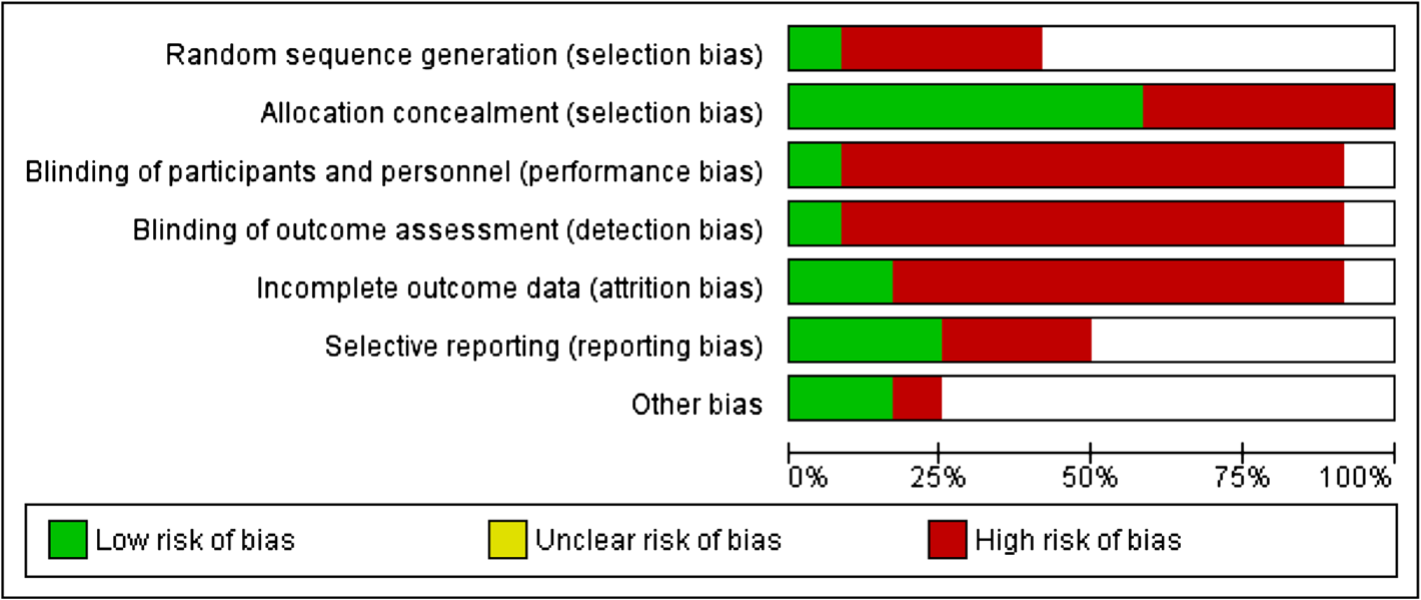
Risk-of-bias graph: reviews the authors’ judgments about each risk-of-bias item presented as percentages across all of the included studies

**Fig. 3:**
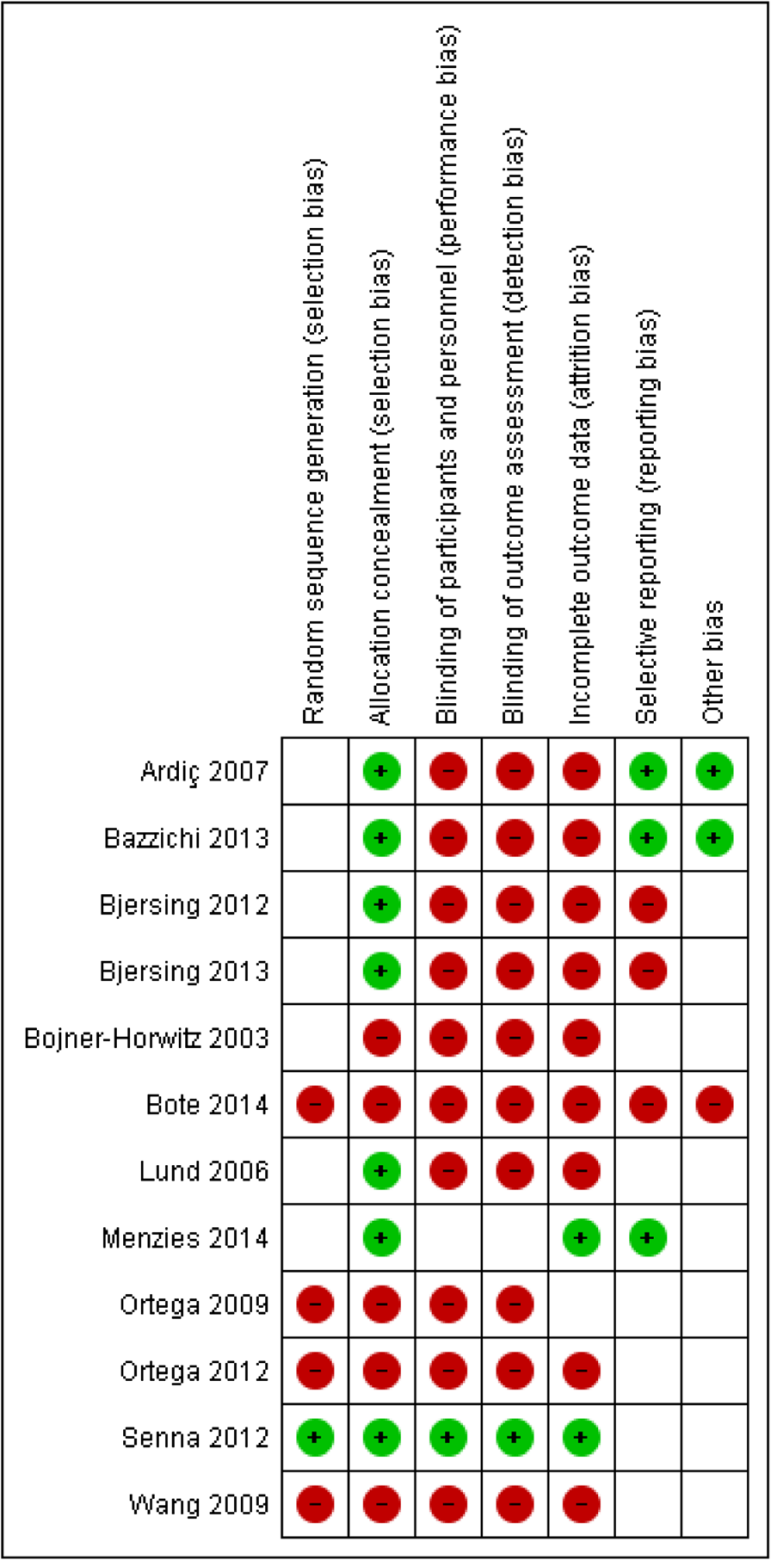
Risk-of-bias summary: review of the authors’ judgments about each risk-of-bias item for each included study

Of all of the included articles, two studies [26, 27] were conducted with almost the same population. With regard to interventions, three types were assessed: complementary and alternative medicine (CAM) (e.g., balneotherapy, massage, mud bath, guided imagery, dance/movement therapy, and dietary therapy), exercise, and multidisciplinary therapy. All of the FM patients were diagnosed by the 1990 ACR criteria [16] (Ortega et al. [28] did not declare the year of the ACR criteria), and the mean age of the participants ranged from 43.4 to 57.0 years old (Ortega et al. [28, 29] did not declare age). With respect to study design, we included eight RCTs, and the remaining four articles included were non-RCTs. According to the Cochrane risk-of-bias tool [25], two studies [30, 31] were considered high quality, but the other trials were low quality with a high risk of bias (Figs. 2 and 3). In addition, of our included trials, only four trials [26, 27, 32, 33] were performed under active control conditions, although two trials [26, 27] were almost the same population. None of these studies included evidence about how the participants rated both the primary and active control conditions as credible, and the likelihood of producing positive results due to baseline intervention expectations was not evaluated. Meanwhile, in relation to the baseline values of each biomarker, our findings revealed that the baseline levels of IL-8 and CRP in FM patients are significantly increased when compared to the healthy control group [28, 34].

### Cytokines

We included nine articles with a total of 440 participants. The characteristics of each study are shown in Table 2. Of all of the studies, 10 of the biomarkers examined were classified as pro-inflammatory cytokines, 4 were classified as anti-inflammatory cytokines, 5 were classified as growth factors and 3 were classified as chemokines. Of the pro-inflammatory cytokines, IL-6 and IL-8 were the most frequently measured in five studies each ([26, 28–31], [26, 28, 31, 34, 35], respectively), and IL-1β and TNF-α were second in three studies each [28, 29, 31]. Of the anti-inflammatory cytokines, IL-10 was measured in three studies [28, 29, 31]. For growth factors, insulin-like growth factor-1 (IGF-1), IGF binding protein-3 (IGFBP3), and nerve growth factor (NGF) were measured in two studies each [26, 27]. Of the chemokines, IL-8 was assessed in five studies [26, 28, 31, 34, 35].

**Table 2:** Characteristics of included trials related to cytokines

Reference	Participants	Study design	Diagnosis of FM	Treatment group	Control group	Study duration	Outcomes	Findings
Ardiç et al. (2007) [37]	N = 31	RCT	ACR 1990	Balneotherapy	Usual care	N/A	IL-1α, CRP, PGE2, LTB4, RF, ESR	In the balneotherapy group, the levels of serum IL-1α significantly decreased after intervention therapy (*p* <0.05)
	Mean age:			(n = 12)	(n = 9)		Two time points	
	43.5 ± 10.2 years (balneotherapy)			3 weeks	3 weeks		(before and at the end of therapy)	
	48.8 ± 8.8 years (usual care)			5 days per week	Healthy		Serum	
	43.4 ± 8.2 years (healthy)			20 minutes each	(n = 10)			
	Gender:							
	female only							
Ortega et al. (2009) [28]	N = 27	Non-RCT (pilot)	ACR	Patients	Healthy	N/A	IL-1β, IL-2, IFN-γ, TNF-α, IL-8, IL-6, IL-4,	After 4 months of exercise, the levels of circulating serum IL-8 and IFN-γ in FM patients decreased significantly (*p* <0.001, *p* <0.05, respectively)
	Age range:		(unspecified year)	(n = 14)	(n = 13)		IL-10, CRP, NA, cortisol	
	30–60 years (patients)			Pool-aquatic exercise			2 time points	
	28–55 years (healthy)			4 months			(before and two days after finishing the exercise)	
	Gender:			3 days per week				
	female only			60 minutes each			serum	
Wang et al. (2009) [34]	N = 100	Non-RCT	ACR 1990	Patients (inpatients)	Healthy	6 months	IL-8	At the beginning of the study, the levels of serum IL-8 in FM patients were significantly higher than the healthy control group (*p* <0.001)
	Mean age:			(n = 20)	(n = 80)		4 time points	
	49.9 ± 6.8 years (patients)			Multidisciplinary therapy			(the beginning of the study, 10 days after	At the end of the study (6 months), the levels of serum IL-8 in FM patients decreased significantly compared to the beginning of the study (*p* <0.05) with a decline to the normal range
	48.4 ± 11.1 years (healthy)			3 weeks			treatment, at resignation (21 days), and at 6	
	Gender:			5 days per week			months follow up)	
	male 15 %, female 85 % (patients)			8-hour session each			serum	
	male 45.2 %, female 54.8 % (healthy)							
Bjersing et al. (2012) [26]	N = 49	RCT	ACR 1990	Nordic walking	Active control: Low-intensity walking	30 weeks	IL-6, IL-8, IGF-1, IGFBP-3, NGF,	There were no significant differences pre- and 15-week post-intervention in the levels of serum IGF-1 and IGFBP-3 between the intervention and active control group
	Mean age:			(n = 26)	(n = 23)		SP, NPY, MMP-3	
	52 years (total)			15 weeks	15 weeks		3 time points: serum IGF-1, IGFBP-3	The change in serum free IGF-1 correlated positively with an alteration in CSF SP levels (*p* <0.1), NPY (*p* = 0.001) and pain threshold (*p* <0.1)
	Gender:			twice a week	twice a week		(baseline, after 15 weeks of exercise, 30	
	female only			40–45 minutes each	40–45 minutes each		weeks of follow up)	
							Two time points: CSF IL-6, IL-8, NGF, SP,	
							NPY, MMP-3	
							(baseline and after 15 weeks of exercise)	
							serum, CSF	
Ortega et al. (2012) [29]	N = 18	Non-RCT	ACR 1990	Patients	Healthy	N/A	IL-1β, TNF-α, IL-6, IL-10, CRP	After 4 months of exercise, the spontaneous and LPS-induced production of IL-6 in FM patients increased and spontaneous TNF-α decreased compared to their basal state
	Age range:			(n = 9)	(n = 9)		3 time points	
	30–60 years (patients)			Pool-aquatic exercise			(before, midway through: 4 months and	
	28–55 years (healthy)			8 months			at the end of program: 8 months)	After 8 months of exercise, the production of IL-1β, TNF-α, IL-6 in FM patients decreased compared to their basal state (both spontaneously and in response to LPS activation), with levels similar to (and even lower than in the case of the spontaneous release of TNF-α) those in the healthy control group
	Gender:			twice a week			(samples were collected 2 days after	
	female only			60 minutes each			finishing the last session of the exercise)	
							serum	
								After 8 months of exercise, the spontaneous production of IL-10 decreased and the LPS-induced production of IL-10 increased compared to their basal state, with levels higher than those in the healthy control group
Senna et al. (2012) [30]	N = 83	RCT	ACR 1990	Dietary weight loss	Usual care	N/A	IL-6, CRP	The levels of serum IL-6 in the intervention group were significantly lower than the usual care group after 6 months of intervention (*p* <0.05), although there were no significant differences between the two groups at baseline.
	Mean age:			(n = 41)	(n = 42)		two time points	
	44.8 ± 13.6 years (intervention)			6 months	6 months		(baseline and after 6 months of intervention)	
	(BMI 32.3 ± 1.4)			1200 kcal/day			serum	
	46.3 ± 14.4 years (usual care)			(with 15–20 % of energy intake in the form of protein, 50–55 % in the form of carbo-hydrates, and approximately 30 % in the form of fat divided across three meals)				
	(BMI 32.8 ± 1.4)							
	Gender:							
	male 9.8 %, female 90.2 % (intervention)							
	male 9.5 %, female 90.5 % (usual care)							
Bjersing et al. (2013) [27]	N = 48	RCT	ACR 1990	Nordic walking	Active control: low-intensity walking	30 weeks	IGF-1, IGFBP3, NGF, adiponectin, leptin, resistin, NPY	In lean patients, the levels of total serum IGF-1 increased after 15 weeks of exercise (*p* <0.05).
	Mean age:			(n = 26)	(n = 22)			
	52.0 years (lean group)			(lean 4, overweight 15, obese 7)	(lean 5, overweight 11, obese 6)		3 time points	The change in the levels of total serum IGF-1 differed significantly between lean and obese patients (*p* = 0.01).
	(BMI 18.5 to 24.9, n = 9)			15 weeks	15 weeks		(baseline, after 15 weeks of exercise, 30	
	53.0 years (overweight group)			twice a week	twice a week		weeks of follow up)	
	(BMI 25 to 29.9, n = 26)			40–45 minutes each	40–45 minutes each		serum, CSF	
	51.0 years (obese group)							
	(BMI ≥30, n = 13)							
	Gender:							
	female only							
Bote et al. (2014) [35]	N = 20	Non-RCT	ACR 1990	Patients	Inactive patients	N/A	IL-8, NA, neutrophils’ function	After 4 months of exercise, there were no significant changes between exercised and non-exercised FM patients in the concentration of serum IL-8
	Mean age:			(n = 10)	(n = 10)		3 time points	
	53 ± 2 years (patients)			Pool-aquatic exercise			(before, midway through: 4 months and	After 8 months of exercise, the concentration of serum IL-8 in exercised FM patients decreased significantly compared to the control group (*p* <0.05).
	50 ± 4 years (inactive patients)			8 months			at the end of program: 8 months)	
	Gender;			twice a week			(samples were collected 2 days after	
	female only (patients)			60 minutes each			finishing the last session of the exercise)	
	not described (inactive patients)						serum, plasma	
Menzies et al. (2014) [31]	N = 64	RCT	ACR 1990	Guided imagery	Usual care	N/A	IFN-γ, TNF-α, IL-1β, IL-2, GM-CSF,	There were no statistically significant differences between the intervention and control groups in the levels of pro- and anti-inflammatory cytokines at baseline, 6 weeks or 10 weeks
	Mean age:			(n = 30)	(n = 34)		IL-12, IL-17, IL-8, MCP-1, MIP-1β, IL-6,	
	44.5 ± 13.1 years (guided imagery)			10 weeks	10 weeks		IL-7, IL-4, IL-5, IL-10, IL-13, G-CSF, CRP	
	49.1 ± 12.4 years (usual care)			Use CD tracks at least once a daily			3 time points	There was a notable trend in the increase of plasma IL-7 in the intervention group, whereas the control group means remained relatively constant across the study interval
	Gender:			For the first 6 weeks, listen to the three			(baseline, week 6, week10)	
	female only			CD tracks (each one CD in two weeks).			plasma	
				For the last 4 weeks, listen to the tracks in any order				

*ACR* American College of Rheumatology, *BMI* body mass index, *CD* compact disc, *CRP* C-reactive protein, *CSF* cerebrospinal fluid, *ESR* erythrocyte sedimentation rate, *G-CSF* granulocyte-colony stimulating factor, *GM-CSF* granulocyte macrophage colony-stimulating factor, *IFN-γ* interferon gamma, *IGFBP-3* insulin-like growth factor-binding protein-3, *IGF-1* insulin-like growth factor-1, *IL* interleukin, *LPS*, lipopolysaccharide, *LTB4* leukotriene B4, *MCP-1* monocyte chemoattractant protein-1, *MIP-1β* macrophage inflammatory protein-1 beta, *MMP-3* matrix metallopeptidase-3, *N/A* not available, *NGF* nerve growth factor, *NPY* neuropeptide Y, *Non-RCT* non-randomized controlled trial, *PGE2* prostaglandin E2, *PRL* prolactin, *RCT* randomized controlled trial, *RF* rheumatoid factor, *SP* surfactant protein, *TNF-α* tumor necrosis factor-alpha

In terms of pro-inflammatory cytokines, three trials [28, 34, 35] observed that the serum levels of IL-8 in FM patients decreased within group between pre-intervention and 4 months post pool-aquatic exercise (*p* <0.001) [28] and 6 months post multidisciplinary therapy (*p* <0.05) [34], and among groups 8 months post pool-aquatic exercise (*p* <0.05) [35]. For IL-6, one trial [29] showed that the spontaneous and lipopolysaccharide (LPS)-induced production of IL-6 in FM patients decreased within group between pre-intervention and 8 months post pool-aquatic exercise (*p* <0.001), although IL-6 levels increased within group between pre-intervention and 4 months exercise (*p* <0.001). In addition, Senna et al. [30] reported that the levels of serum IL-6 in the dietary therapy group decreased within group between baseline and post 6 months of intervention.

With regard to anti-inflammatory cytokines, the findings were inconsistent. Ortega et al. [29] demonstrated that the spontaneous production of IL-10 in FM patients decreased within group between pre-intervention and 8 months post pool-aquatic exercise (*p* <0.001), whereas LPS induced IL-10 to increase (*p* <0.05) within group. With respect to growth factors, Bjersing et al. [27] showed that the levels of total serum IGF-1 in lean FM patients increased within group after 15 weeks of Nordic walking (*p* <0.05).

### Neuropeptides

We included five articles with a total of 193 participants. Of these articles, two studies [26, 27] were also included in the group that utilized cytokines and were conducted with almost the same population. The characteristics of each study are shown in Table 3. Of all of the studies, nine of the biomarkers examined were neuropeptides. Neuropeptide Y (NPY) was measured in three studies [26, 27, 36], and the others were assessed in one study each.

**Table 3:** Characteristics of included trials related to neuropeptides

Reference	Participants	Study design	Diagnosis of FM	Treatment group	Control group	Study duration	Outcomes	Findings
Bojner-Horwitz et al. (2003) [36]	N = 36	RCT	ACR 1990	Dance/movement	Waiting control	14 months	PRL, NPY, DHEA-S, cortisol	The levels of serum PRL in both groups increased across the study interval, with the largest increase in the intervention group
	Mean age:			(n = 20)	(n = 16)		4 time points (at baseline, and at months 4, 6, and 14 of the study)	
	57 ± 7.2 years (total)			6 months	6 months			There were no significant differences between baseline and 14 months in the levels of serum PRL between the two groups
	Gender:			once a week				
	female only			1 h each			serum, saliva	The levels of serum NPY in both groups increased from baseline to month 4, decreased from months 4 − 6, and increased from months 6 − 14
								There were no significant differences in the levels of serum NPY between the two groups across the study interval
Lund et al. (2006) [32]	N = 19	RCT	ACR 1990	Massage	Guided relaxation	10 weeks	CRF-LI	In the massage group, the concentrations of urinary CRF-LI decreased after 6 weeks of massage treatment (*p* = 0.01) and 1 month after completion of treatments (*p* <0.5)
	Mean age:			(n = 10)	(n = 9)		3 time points (prior to treatment, after 6-week treatment and 1 month after completed treatment) urine	
	50.7 ± 9.7 years (total)			6 weeks	6 weeks			
	Gender:			twice weekly	twice weekly			
	f+emale only			30 minutes each (feet and legs 18 minutes, hands and arm 8 minutes, face 4 minutes)	30 minutes each			
Bjersing et al. (2012) [26]	N = 49	RCT	ACR 1990	Nordic walking	Active control: low-intensity walking	30 weeks	SP, NPY,	The change in the levels of serum IGF-1 correlated positively with alterations in CSF SP (*p* <0.1), NPY (*p* <0.01) and pain threshold (*p* <0.1)
	Mean age:			(*N* = 26)	(n = 23)		IL-6, IL-8, IGF-1, IGFBP3, NGF, MMP-3	
	52 years (total)			15 weeks	15 weeks		3 time points	
	Gender:			twice a week	twice a week		(baseline, after 15 weeks of exercise, 30 weeks of follow up)	
	female only			40–45 minutes each	40–45 minutes each			
							serum, CSF	
Bazzichi et al. (2013) [33]	N = 41	RCT	ACR 1990	Balneotherapy	Active control: mud-bath therapy	12 weeks	BDNF, oxytocin,	The concentrations of serum BDNF significantly decreased in both balneo-therapy and mud-bath therapy after 12 weeks (*p* < 0.05, *p* < 0.01, respectively), while there was no significant change in the levels of oxytocin.
	Mean age:			(n = 20)	(n = 21)		SERT binding parameters, ATP	
	54.0 ± 7.2 years (balneotherapy)			2 weeks	2 weeks		3 time points	
	52.8 ± 10.2 years (mud-bath therapy)			6 days a week	6 days a week		(at baseline, after 2 weeks and after 12 weeks)	
	Gender:			20 minutes each	20 minutes each (mud pack 10 minutes and immersion in thermal water 10 minutes)			
	19/1 female/male (balneotherapy)						plasma, serum, salivary adiponectin, leptin, resistin, NPY, IGF-1, IGFBP3, NGF	
	20/1 female/male (mud-bath therapy)							
Bjersing et al. (2013) [27]	N = 48	RCT	ACR 1990	Nordic walking	Active control: low-intensity walking	30 weeks		The levels of serum resistin increased in the group as a whole after 30 weeks (*p* <0.05) which correlated with decreased fatigue
	Mean age:			(n = 26)	(n = 22)			
	52.0 years (lean group)			(lean 4, overweight 15, obese 7)	(lean 5, overweight 11, obese 6)		3 time points	The levels of serum NPY increased in the group as a whole after 30 weeks (*p* <0.05). This increase was only significant in obese patients (*p* <0.05)
	(BMI 18.5 to 24.9, n = 9)			15 weeks	15 weeks		(baseline, after 15 weeks of exercise, 30 weeks of follow up) serum, CSF	
	53.0 years (overweight group)			twice a week	twice a week			
	(BMI 25 to 29.9, n = 26)			40–45 minutes each	40–45 minutes each			
	51.0 years (obese group)							
	(BMI ≥30, n = 13)							
	Gender:							
	female only							

*ACR* American College of Rheumatology, *ATP* adenosine 5′-triphosphate, *BDNF* brain-derived neurotrophic factor, *BMI* body mass index, *CRF-L1* corticotropin releasing factor-like immunoreactivity, *CSF* cerebrospinal fluid, *DHEA-S* dehydroepiandrosterone sulfate, *IGFBP-3* insulin-like growth factor-binding protein-3, *IGF-1*, insulin-like growth factor-1, *IL* interleukin, *MMP-3* matrix metallopeptidase-3, *NGF* nerve growth factor, *Non-RCT* non-randomized controlled trial, *NPY* neuropeptide Y, *PRL* prolactin, *RCT* randomized controlled trial, *SERT* serotonin transporter, *SP* surfactant protein

For NPY, two trials [27, 36] demonstrated no consistent findings. Bjersing et al. [27] demonstrated that the levels of serum NPY in obese FM patients significantly increased within group after 30 weeks (including 15-week exercise and 15-week follow up) (*p* <0.05). By contrast, Bojner-Horwitz et al. [36] reported that the levels of serum NPY in both the dance/movement group and the control group increased compared to baseline and month 14 of the study within each group.

In terms of other neuropeptides, the concentrations of urinary corticotropin releasing factor-like immunoreactivity (CRF-L1) in the massage group decreased within group after 6 weeks of massage treatment (*p* = 0.01) and 1 month after completed treatments (*p* <0.5) [32]. For brain-derived neurotrophic factor (BDNF), Bazzichi et al. [33] noted that the concentrations of serum BDNF significantly decreased within each group in both the 2-week balneotherapy group and the 2-week mud bath therapy group after 12 weeks (*p* <0.05, *p* <0.01, respectively). Furthermore, for resistin, Bjersing et al. [27] demonstrated that the levels of serum resistin in the group as a whole increased within group after 30 weeks (including 15 weeks of exercise and 15 weeks of follow up) (*p* <0.05), which correlated with decreased fatigue.

### CRP

We included five articles with a total of 223 participants. All of these articles [28–31, 37] were also included in the group that utilized cytokines. The characteristics of each study are shown in Table 4.

**Table 4:** Characteristics of included trials related to CRP

Reference	Participants	Study design	Diagnosis of FM	Treatment group	Control group	Study duration	Outcomes	Findings
Ardiç et al. (2007) [37]	N = 31	RCT	ACR 1990	Balneotherapy	Usual care	N/A	CRP, IL-1α, PGE2, LTB4, RF, ESR	No declaration of the changes to serum CRP levels between pre- and post-intervention
	Mean age:			(n = 12)	(n = 9)		2 time points	
	43.5 ± 10.2 years (balneotherapy)			3 weeks	3 weeks		(before and at the end of therapy)	
	48.8 ± 8.8 years (usual care)			5 days per week	Healthy		serum	
	43.4 ± 8.2 years (healthy)			20 minutes each	(n = 10)			
	Gender:							
	female only							
Ortega et al. (2009) [28]	N = 27	Non-RCT (pilot)	ACR	Patients	Healthy	N/A	CRP, IL-1β, IL-2, IFN-γ, TNF-α, IL-8,	The concentrations of serum CRP in FM patients were significantly higher than in the healthy control group between pre- and post-exercise (*p* <0.05)
	Age range:		(unspecified year)	(n = 14)	(n = 13)		IL-6, IL-4, IL-10, NA, cortisol	
	30–60 years (patients)			Pool-aquatic exercise			2 time points	After 4 months of exercise, the level of serum CRP in FM patients decreased compared to the baseline level (*p* <0.05)
	28–55 years (healthy)			4 months			(before and 2 days after finishing the exercise)	
	Gender:			3 days per week				
	female only			60 minutes each			serum	
Ortega et al. (2012) [29]	N = 18	Non-RCT	ACR 1990	Patients	Healthy	N/A	CRP, IL-1β, TNF-α, IL-6, IL-10	The concentrations of serum CRP in FM patients were significantly higher than in the healthy control group across the study interval (*p* <0.01)
	Age range:			(n = 9)	(n = 9)		3 time points	
	30–60 years (patients)			Pool-aquatic exercise			(before, midway through: 4 months and at the end of program: 8 months)	After 8 months of exercise, the level of serum CRP in FM patients decreased compared to the baseline level (*p* <0.05)
	28–55 years (healthy)			8 months				
	Gender:			twice a week			(samples were collected 2 days after finishing the last session of the exercise) serum	
	female only			60 minutes each				
Senna et al. (2012) [30]	N = 83	RCT	ACR 1990	Dietary weight loss	Usual care	N/A	CRP, IL-6	The levels of serum CRP in the intervention group were significantly lower than the usual care group after 6 months of intervention (*p* <0.01), although there were no significant differences between the two groups at baseline
	Mean age:			(*N* = 41)	(n = 42)		2 time points	
	44.8 ± 13.6 years (intervention)			6 months	6 months		(baseline and after 6 months of intervention)	
	(BMI 32.3 ± 1.4)			1200 kcal/day			serum	
	46.3 ± 14.4 years (usual care)			(with 15–20 % of energy intake in the form of protein, 50–55 % in the form of carbo-hydrates, and approximately 30 % in the form of fat divided in three meals)				
	(BMI 32.8 ± 1.4)							
	Gender:							
	male 9.8 %, female 90.2 % (intervention) male 9.5 %, female 90.5 % (usual care)							
Menzies et al. (2014) [31]	N = 64	RCT	ACR 1990	Guided imagery	Usual care	N/A	CRP, IFN-γ, TNF-α, IL-1β, IL-2, GM-	There were no statistically significant differences between the intervention and control groups in the levels of plasma CRP at baseline, 6 weeks or 10 weeks
	Mean age:			(n = 30)	(n = 34)		CSF, IL-12, IL-17, IL-8, MCP-1, MIP-1β,	
	44.5 ± 13.1 years (guided imagery)			10 weeks	10 weeks		IL-6, IL-7, IL-4, IL-5, IL-10, IL-13, G-CSF	The levels of plasma CRP for all of the participants were elevated but demonstrated little variation from baseline to 6 weeks or to 10 weeks (4.27, 4.57, 4.55 mg/L, respectively)
	49.1 ± 12.4 years (Usual care)			use CD tracks at least once a day			3 time points	
	Gender:			For the first 6 weeks, listen to the three			(baseline, week 6, week 10) plasma	
	female only			CD tracks (each one CD in 2 weeks).				
				For the last 4 weeks, listen to the tracks in any order				

*ACR* American College of Rheumatology, *BMI* body mass index, *CD* compact disc, *CRP* C-reactive protein, *ESR* erythrocyte sedimentation rate, *G-CSF* granulocyte-colony stimulating factor, *GM-CSF* granulocyte macrophage colony-stimulating factor, *IFN-γ* interferon gamma, *IL* interleukin, *LTB4* leukotriene B4, *MCP-1* monocyte chemoattractant protein-1, *MIP-1β* macrophage inflammatory protein-1 beta, *NA* noradrenaline, *N/A* not available, *Non-RCT* non-randomized controlled trial, *PGE2* prostaglandin E2, *RCT* randomized controlled trial, *RF* rheumatoid factor, *TNF-α*, tumor necrosis factor-alpha

Of these studies, Ardiç et al. [37] did not observe changes in serum CRP levels between pre- and post-balneotherapy intervention. Three trials [28–30] observed that the levels of serum CRP in the intervention group decreased within group compared to the baseline level after 4 months or 8 months of pool-aquatic exercise (*p* <0.05, each) [28, 29] and 6 months of dietary therapy [30]. Contrary to these trials, the remaining trial [31] showed that there were no statistically significant differences between the guided imagery group and the group receiving typical care in the levels of plasma CRP at baseline, 6 weeks or 10 weeks. Comparing the levels of CRP at baseline, three trials [28, 29, 31] indicated that the levels of CRP in FM patients were higher than the reference value.

## Discussion

We aimed to assess the effects of non-pharmacological interventions on biomarkers (specifically cytokines, neuropeptides, and CRP) in patients with FM. Despite the importance of non-pharmacological interventions in FM patients, few studies have focused on changes to biomarkers after non-pharmacological intervention. In fact, to our knowledge, this is the first systematic review on this subject. We found only 12 articles that fulfilled our inclusion criteria. Of these articles, only one trial examined psychological interventions [31].

### Cytokines

Our findings indicated that the levels of serum IL-8 in FM patients decreased after exercise or multidisciplinary interventions and those of IL-6 (spontaneous, LPS-induced, or serum) in FM patients tended to decrease after exercise or dietary interventions. For IL-8, three trials revealed a reduction in serum IL-8 levels between pre- and post-intervention, including 3 weeks of multidisciplinary exercise or 4 or 8 months of pool-aquatic exercise [28, 34, 35]. IL-8 is both a pro-inflammatory cytokine that activates neutrophils and a chemokine that plays an important role in the infiltration of neutrophils at inflammation sites [22]. For IL-6, one trial demonstrated a reduction of the spontaneous and LPS-induced production of IL-6 between pre-intervention and 8 months post exercise within group [29]. Moreover, another trial showed that the levels of serum IL-6 in the dietary therapy group decreased between baseline and after 6 months of intervention within group [30]. Taking into consideration these findings, exercise, in particular, could act as an anti-inflammatory influence in FM patients, although it could act as a pro-inflammatory influence in healthy individuals [21]. In other words, exercise interventions could improve the inflammatory status in FM patients, reaching values close to the basal levels in the healthy population, by adjusting the inflammatory-stress feedback mechanism [21].

In terms of anti-inflammatory cytokines, however, our findings were inconsistent. Ortega et al. [29] reported that the spontaneous production of IL-10 in FM patients decreased between pre-intervention and 8 months post exercise within group, whereas LPS-induced IL-10 to increase within group. It remains unclear if this discrepancy is related to only the diverse functions of IL-10 and other factors, or the smaller sample size.

In addition, with regard to growth factors, Bjersing et al. [27] demonstrated that the levels of total serum IGF-1 in lean FM patients increased after 15 weeks of exercise within group, whereas these levels were unchanged in overweight or obese FM patients within each group. They also found evidence of a positive influence of IGF-1 on fatigue. Given that the fatigue response exercise is related to levels of body mass index (BMI), changes in IGF-1 might indicate a beneficial role for fatigue in obese FM patients.

### Neuropeptides and CRP

We found no consistent changes in the levels of neuropeptides or CRP due to non-pharmacological interventions. One trial [27] reported that the levels of serum NPY in obese FM patients significantly increased after 30 weeks (including 15 weeks of exercise and a 15-week follow-up) (*p* <0.05) within group, whereas in the other trial [36] they increased both in the dance/movement group and the control group compared to baseline after 14 months treatment, within each group.

With respect to other neuropeptides, however, some notable results have been observed, although only one trial was conducted to study each neuropeptide. Bjersing et al. [27] also reported that the levels of serum resistin in all FM patients increased after 30 weeks (including 15 weeks of exercise and 15-week follow up) within group, which correlated with decreased fatigue. In view of the abovementioned results, they noted that IGF-1 and resistin were involved in the mechanism that reduced fatigue after moderate exercise in FM patients.

Of the other trials, Lund et al. [32] demonstrated that the concentrations of urinary CRF-LI in the massage group decreased after 6 weeks of massage treatment and 1 month after completion of the treatments, within group. Furthermore, Bazzichi et al. [33] reported that the concentrations of serum BDNF significantly decreased in both 2-week balneotherapy and 2-week mud bath therapy after 12 weeks, within each group. Although these two trials [32, 33] were not exercise intervention but manipulative and body-based non-pharmacological interventions, CRF-L1 and BDNF could be used as indicators of the effects of each intervention in FM patients.

In terms of CRP, as with the neuropeptides, our findings were inconsistent. In three trials [28–30] the levels of serum CRP in the intervention group decreased compared to the baseline level after 4 or 8 months of pool-aquatic exercise (*p* <0.05, each), within group [28, 29] or 6 months of dietary therapy within group [30]. In contrast, another trial [31] noted that there were no significant differences in the levels of plasma CRP between the guided imagery group and the group receiving usual care at baseline, 6 weeks or 10 weeks. Further research comparing each non-pharmacological intervention, specifically psychological and non-psychological intervention, is required to clarify the changes in CRP levels in FM patients.

### Limitations

The results of this review have several limitations. The primary limitation is the paucity and low quality of the included studies. We only included 12 articles (two trials were conducted with almost the same population), and of these, there were only 8 RCTs. The quality of the included trials has been assessed with the Cochrane risk-of-bias tool [25], which showed that only two studies [30, 31] were considered high quality, whereas the others were low quality with a high risk of bias. With regard to publication bias, we did not conduct the investigation using funnel plot analysis due to the high levels of heterogeneity among studies, and small sample sizes [38], and unfortunately we were unable to make any inference from this.

Another limitation is the high heterogeneity of non-pharmacological interventions. They consisted of three types: CAM, exercise, and multidisciplinary therapy. Of these types of treatment, CAM was also divided into the following categories: mind-body therapies (e.g., guided imagery, dance/movement therapy), manipulative and body-based therapy (e.g., balneotherapy, massage, mud-bath), and biological-based therapies (e.g., dietary therapy) [39]. Furthermore, the duration of each intervention and each study had various patterns. In particular paying attention to follow up, of our included trials, six [26, 27, 32–34, 36] were conducted with a follow-up period, although two [26, 27] were based on almost the same population. Of these six trials, only two [32, 34] demonstrated that the effect of each intervention in the levels of biomarkers continued over the follow-up period within the intervention group. Lund et al. [32] showed that the concentrations of urinary CRF-L1 decreased over the 10-week study duration within the intervention group, and Wang et al. [34] reported that the levels of serum IL-8 decreased over the 6-month study duration within the intervention group. On the other hand, although we only included the studies with non-pharmacological interventions, there are few studies on the effect of pharmacological treatments in inflammatory biomarkers in FM, whereas it appears that antidepressants can normalize levels of some biomarkers such as ACTH [19]. Our results for each intervention should be interpreted carefully.

An additional limitation is the high variability of materials. Biomarkers were investigated in diverse materials: serum, plasma, cerebrospinal fluid (CSF), urine, and saliva. Thus, a direct comparison of the results is hardly possible even when the same biomarker was compared.

Moreover, it is unclear whether our findings are exclusive to FM patients [21]. Of all of our included studies, only FM patients or FM patients and healthy subjects were included. The non-pharmacological interventions that were conducted in our selected articles might affect other inflammatory diseases. Ploeger et al. [40] reported that the inflammatory response in patients with chronic inflammatory diseases compared to the healthy population generally increased after acute exercise. Further research is needed to elucidate whether non-pharmacological interventions are effective only for patients with FM.

In addition, there are not enough studies on the effect of non-pharmacological interventions on inflammatory biomarkers in healthy individuals, and we are unable to compare these effects and be certain that the effects reported here are related to FM. For instance, exercise could act as an anti-inflammatory influence in FM patients, whereas it could act as a pro-inflammatory influence in healthy individuals [21]. Further research is required to explore whether the effects of non-pharmacological interventions on inflammatory biomarkers are different in FM patients compared to the healthy population.

### Future directions

Our findings, above all else, highlight the need to conduct further RCTs focused on biomarker changes after non-pharmacological interventions in FM patients. The following provisions would be helpful to improve future research in FM patients. First, non-pharmacological interventions should include not only exercise but also psychological treatment. Only one trial, guided imagery has been conducted as a psychological intervention [31]. Of note, no study has assessed the efficacy of mindfulness on biomarkers except for cortisol [41] in FM patients. We need to elucidate the relationships between psychological interventions and biomarkers in patients with FM.

Second, biomarkers should be measured under the same conditions (e.g., controlling for menstrual phase and medications) to obtain homogeneous patient samples. As recommended in RCTs, control groups should be randomized and active, using standardized low-intensity non-pharmacological interventions (and not waiting list or non-active interventions). Follow up is mandatory and standardized periods should be accepted by researchers (for instance: post-treatment, 3-month and 12-month follow up). In addition, more neuropeptides should be included as biomarkers. Finally, the relationship between these biomarkers and pain-related measures deserves to be studied. Third, participants should be homogeneous, specifically for disease duration and age. Given that many of FM patients are female, more attention should be paid to the effects of menstruation. Moreover, participants who do not regularly take some kinds of medications (e.g., analgesic or psychotropic drugs) would be desirable. Future trials should be conducted with the aim of reducing the influence of these medications. On the other hand, participants would include not only patients with FM but also patients with other inflammatory diseases. Few studies have focused on comparing the effects of non-pharmacological interventions between FM and other inflammatory diseases.

## Conclusions

In conclusion, our results suggest that the levels of serum IL-8 in the patients with FM decreased after exercise or multidisciplinary interventions, and IL-6 levels (spontaneous, LPS-induced, or serum) in FM patients tended to decrease after exercise or dietary interventions. Exercise intervention, in particular, could act as an anti-inflammatory influence in FM patients, although it might act as a pro-inflammatory influence in a healthy population. To summarize, exercise interventions could ameliorate the inflammatory status of FM patients, generating values closer to the baseline levels of healthy individuals by regulating the inflammatory-stress feedback mechanism. However, our findings revealed discrepancies in the anti-inflammatory cytokines, but the changes in IGF-1 might indicate a beneficial role for fatigue in obese FM patients.

The results for neuropeptides and CRP seem to be inconsistent. Although only one trial was conducted on resistin, there was an increase in the levels of serum resistin in FM patients after an exercise period, which correlated with decreased fatigue. IGF-1 and resistin might be involved in the effects that reduce fatigue in FM patients. Furthermore, CRF-L1 and BDNF could be used as indicators of efficacy related to non-pharmacological interventions in FM patients.

## Abbreviations

ACR: American College of Rheumatology
BDNF: brain-derived neurotrophic factor
BMI: body mass index
CAM: complementary and alternative medicine
CFS: chronic fatigue syndrome
CRF-L1: corticotropin releasing factor-like immunoreactivity
CRP: C-reactive protein
CSF: cerebrospinal fluid
FM: fibromyalgia
IGF-1: insulin-like growth factor 1
IGFBP3: IGF binding protein-3
IL: interleukin
LPS: lipopolysaccharide
NGF: nerve growth factor
NPY: neuropeptide Y
PRISMA: Preferred Reporting Items for Systematic Reviews and Meta-Analysis guidelines
RCT: randomized controlled trials
TNF-α: tumor necrosis factor-alpha
